# Specialism and Hospital Committees

**Published:** 1914-03-14

**Authors:** 


### The Hospital

The Workers' Newspaper of Administrative Medicine and Institutional Life, Administration, National Insurance and Health.

No. 1446, Vol. LV. SATURDAY, MARCH 14, 1914. PRICE ONE PENNY.

### SPECIALISM AND HOSPITAL COMMITTEES.

There is a large town in a colliery district, with thickly populated environs, possessing a hundred-bed hospital, which from time to time has been duly extended and brought up to date in equipment. Every facility now exists there which a first-rate general surgeon could desire, and also some provision for special work. And, as a fact, difficult surgical cases receive adequate treatment—not, however, from members of the staff, but from surgeons of the nearest provincial city, these gentlemen being called in for the purpose from time to time. This state of affairs, like many another one, appears less strange after a little examination than it does at first sight. There is undoubtedly sufficient population to give a keen young local practitioner opportunity to make a name as a surgeon. But then the town is not attractive residentially; in places of equal size with greater amenities, juniors quite good enough for a large hospital staff (but lacking money with which to sit down and wait) may no doubt be found who act as their own consultants in any surgical matter. But in whatever way the situation has arisen, one officer, at any rate, of the institution under notice seems to find it undesirable, because he has a proposal to make for the purpose of remedying it.

This proposal is that the old rural infirmary plan of allotting indiscriminately medical and surgical cases to each member of the full staff should be dropped, and that for the future patients and doctors both should be divided, so that medical cases go to physicians, and surgical ones to surgeons; with the corollary, reasonable enough, of separate wards. The last requisite could be easily provided, and, of course, the whole change is one bound to occur at a certain, and not very advanced, stage of refinement of institutional care of the sick. What may be considered to weaken the proposal is the fact, however, that its author wishes to be not a surgeon, but a physician. His colleagues object, and perhaps not unnaturally. For if the new plan be brought into operation with the staff as it is at present, some will be left with, perhaps it can scarcely be called the reproach, of requiring outside help, while at the same time the remainder will secure the prestige, rather useful in general practice, of exclusive medical experience. This is how matters might and would appear to the lay eye, but the esoteric view will be different. Nowadays, the general physician, no matter how highly placed, is as an actual authority quite in the background.

We listened the other day to an address by one such on the subject of hæmorrhage, and it was remarkable how for his diagnoses he was dependent upon specialists concerned with the different anatomical regions affected. Internal medicine—what the surgeons have left of it—is visibly breaking up more and more into specialties. Cardiology of itself demands a good deal of study, which (or so it is hinted) some not very senior men are failing to bestow upon this subject. There is now, in Great Britain also, an institution exclusively devoted to the diseases of nutrition. Tuberculosis has a literature which must take a "whole-timer's" efforts to keep up with. Above all, the bacteriologist, as Sir Almroth Wright's recent papers on pneumonia show yet again, has very much to teach to his none too eager pupil, the general medical clinician. Nor is the general surgeon quite untouched. Urologists, at any rate, can boast of an international association, while pure anatomists have been demanding, with reason, greater representation on examining boards for the higher surgical diplomas.

The fact is that increasing specialism, in spite of derision overt and covert, is necessary to the progress of medicine, failing a supply of practitioners each with a mind like Aristotle's. Time shows that the protesters are merely being pushed from pillar to post. Let us put the case to a *laudator temporis acti* that he have the misfortune to swallow incompletely his false teeth. Will he let the general surgeon go to work with a probang like a chimney-sweep, or make a maiden effort with an œsophagoscope, or will he go straight to a laryngologist? And if, as we anticipate, he allows us a point scored here, he must in fairness allow also that all through medicine the old, easy-going, general purview is going out of date and yielding to something a good deal more *eingehend*. Charles Bovary failed with his operation on the cripple, not because he was a general practitioner, but because he was a fool. But now the cleverest man in general consultant medical practice could be set many tasks, nominally within his province and his powers, in which he would fail just as badly. It becomes, therefore, the duty of hospital committees to appreciate all such facts, and to arrange a tactful policy in accordance with the indications proceeding from them.

